# Pairing Voluntary Movement and Muscle-Located Electrical Stimulation Increases Cortical Excitability

**DOI:** 10.3389/fnhum.2016.00482

**Published:** 2016-09-28

**Authors:** Mads Jochumsen, Imran K. Niazi, Nada Signal, Rasmus W. Nedergaard, Kelly Holt, Heidi Haavik, Denise Taylor

**Affiliations:** ^1^Department of Health Science and Technology, Center for Sensory-Motor Interaction, Aalborg UniversityAalborg, Denmark; ^2^Health and Rehabilitation Research Institute, Auckland University of TechnologyAuckland, New Zealand; ^3^Center for Chiropractic Research, New Zealand College of ChiropracticAuckland, New Zealand

**Keywords:** electrical stimulation, motor cortical activation, transcranial magnetic stimulation, motor evoked potentials, corticospinal excitability, neuronal plasticity, long-term potentiation

## Abstract

Learning new motor skills has been correlated with increased cortical excitability. In this study, different location of electrical stimulation (ES), nerve, or muscle, was paired with voluntary movement to investigate if ES paired with voluntary movement (a) would increase the excitability of cortical projections to tibialis anterior and (b) if stimulation location mattered. Cortical excitability changes were quantified using motor evoked potentials (MEPs) elicited by transcranial magnetic stimulation (TMS) at varying intensities during four conditions. Twelve healthy subjects performed 50 dorsiflexions at the ankle during nerve or muscle ES at motor threshold (MTh). ES alone was delivered 50 times and the movement was performed 50 times. A significant increase in the excitability from pre- to post-intervention (*P* = 0.0061) and pre- to 30 min post-intervention (*P* = 0.017) measurements was observed when voluntary movement was paired with muscle ES located at tibialis anterior. An increase of 50 ± 57 and 28 ± 54% in the maximum MEPs was obtained for voluntary movement paired with muscle-located and nerve-located ES, respectively. The maximum MEPs for voluntary movement alone and muscle-located ES alone were −5 ± 28 and 2 ± 42%, respectively. Pairing voluntary movement with muscle-located ES increases excitability of corticospinal projections of tibialis anterior in healthy participants. This finding suggests that active participation during muscle-located ES protocols increases cortical excitability to a greater extent than stimulation alone. The next stage of this research is to investigate the effect in people with stroke. The results may have implications for motor recovery in patients with motor impairments following neurological injury.

## Introduction

Electrical stimulation (ES) has been used to artificially increase cortical excitability with the intent of inducing cortical plasticity. Changes in cortical excitability have been associated with motor skill learning (Pascual-Leone et al., 1995; Thompson and Stein, 2004; Everaert et al., 2010), and with the recovery of movement following stroke (Popovic et al., 2003; Kimberley et al., 2004). It has been reported that afferent feedback is important in influencing cortical excitability (Ridding and Rothwell, 1999; Khaslavskaia et al., 2002) which in turn influences motor skill acquisition (Pavlides et al., 1993). Possible mechanisms for changes in cortical excitability, as a result of afferent inflow and learning, include a reduction of cortical inhibitory circuits and modulation of activity-dependent plasticity leading to long-term potentiation/depression-like (LTP/LTD) plastic changes (Ridding and Rothwell, 1999; Ziemann et al., 2004). In previous studies, it has been reported that cortical excitability may be increased when repetitive ES or functional ES is delivered during or triggered by a voluntary movement (Khaslavskaia and Sinkjaer, 2005; Barsi et al., 2008; Taylor et al., 2012). Voluntary movement activates the cortex by decreasing intracortical inhibition and increases intracortical excitability (Ridding et al., 1995; Classen et al., 1998; Schubert et al., 2004; Taylor et al., 2012). The pairing of motor cortical activity with ES has been outlined in a classical protocol called paired associative stimulation (PAS) (Stefan et al., 2000). The effect of inducing neural plasticity with PAS has been tested in several studies (Stefan et al., 2000; Wolters et al., 2003; Mrachacz-Kersting et al., 2007; Kumpulainen et al., 2012). Cortical activation in PAS protocols is obtained using transcranial magnetic stimulation (TMS). This cortical activation is carefully timed with a single pulse of ES from a muscle of interest. The inter-stimulus interval (ISI) between the peripheral ES and the cortical TMS was shown to be crucial. By altering the ISI, both LTP-like and LTD-like effects have been observed (Stefan et al., 2000; Wolters et al., 2003; Mrachacz-Kersting et al., 2007; Kumpulainen et al., 2012). However, PAS has several problems. TMS generates activation of many motor neuron pools simultaneously, including agonist and antagonist muscles. Consequently the descending corticospinal volley resulting from a single magnetic stimulus consists of multiple waves, due to both direct and indirect activation of corticospinal neurons; thus it is different from normal voluntary movements (Day et al., 1989; Berardelli et al., 1990; Thompson et al., 1991). In terms of the utility of the PAS protocol for people with stroke it is limited by the requirement to use TMS (Rossi et al., 2009). Whether used as an outcome measure or an intervention, TMS must be used cautiously in the stroke population due to its risk of inducing seizures, interfering with implanted devices or metalware, or causing discomfort, headache or syncope (Rossi et al., 2009). To overcome these issues, it was hypothesized that single pulse peripheral ES could be paired with voluntary movement. This pairing can be realized by the detection of movement-related cortical potential (MRCP) associated with movement planning and execution (Mrachacz-Kersting et al., 2012; Niazi et al., 2012).

The MRCP is a brain potential associated with the planning and execution of real and imagined voluntary movements and can be extracted from electroencephalography (EEG) (Nascimento et al., 2006). Similar to conventional PAS protocols, in the MRCP PAS protocol the timing of the afferent volley from the ES and the cortical activation is crucial. It has been reported that the afferent volley should coincide with the most negative part of the MRCP (where the cortical activation of the areas associated with the upcoming movement is highest) (Mrachacz-Kersting et al., 2012). When timed appropriately increased cortical excitability was found following 50 pairings of the peak negativity of the MRCP (from imaginary movements) with single pulse ES. This supports the notion that the pairing of the MRCP with the ES is the component that results in increased cortical activation. Executed and imaginary movements have shown to lead to increases in cortical excitability (Pascual-Leone et al., 1995; Khaslavskaia and Sinkjaer, 2005; Stinear et al., 2006), but for 50 repetitions no changes were observed in the cortical excitability (Mrachacz-Kersting et al., 2012). In this study ES located at the muscle is proposed as an alternative to ES located over a peripheral nerve, which may be more comfortable for patients particularly during relatively high repetitions of stimulation (at least 50 stimulation pairs). A risk of using ES located at the muscle is that the afferent input may be attenuated as stimulation frequency is increased, as is observed for somatosensory evoked potentials (SEPs) through sensory gating, potentially from activation of secondary afferents (Fujii et al., 1994; Mrachacz-Kersting and Sinkjaer, 2008). However, the conclusions of the functional implications of sensory gating based on SEPs must be taken with caution (Morita et al., 1998); attenuated amplitudes of SEPs are also seen during normal voluntary movements (Starr and Cohen, 1985; Chéron and Borenstein, 1987; Cohen and Starr, 1987; Rossini et al., 1996; Tinazzi et al., 1997). ES located over a muscle may be a good candidate for the induction of Hebbian-associated plasticity based on the persistent activation of neural circuits (Hebb, 1949; Thickbroom, 2007).

The aims of this study were: (a) to investigate whether the excitability of the cortical projections of tibialis anterior (TA) increased when combining a voluntary dorsiflexion of the ankle joint with ES, (b) if there were differences when using muscle-located ES instead of nerve-located ES. Also muscle ES alone and ME alone were assessed as conditions.

## Materials and methods

### Subjects

Twelve healthy subjects participated in this study, 5 males and 7 females (mean age: 27 ± 3 years). All subjects filled in a screening questionnaire for TMS eligibility based on the recommendation in Rossi et al. (2009) and signed a consent form before participation. All procedures were approved by the local ethical committee (14/NTB/113) according to the Helsinki declaration.

### Voluntary movement and stimulation

#### Voluntary movement

**Figure 1:**
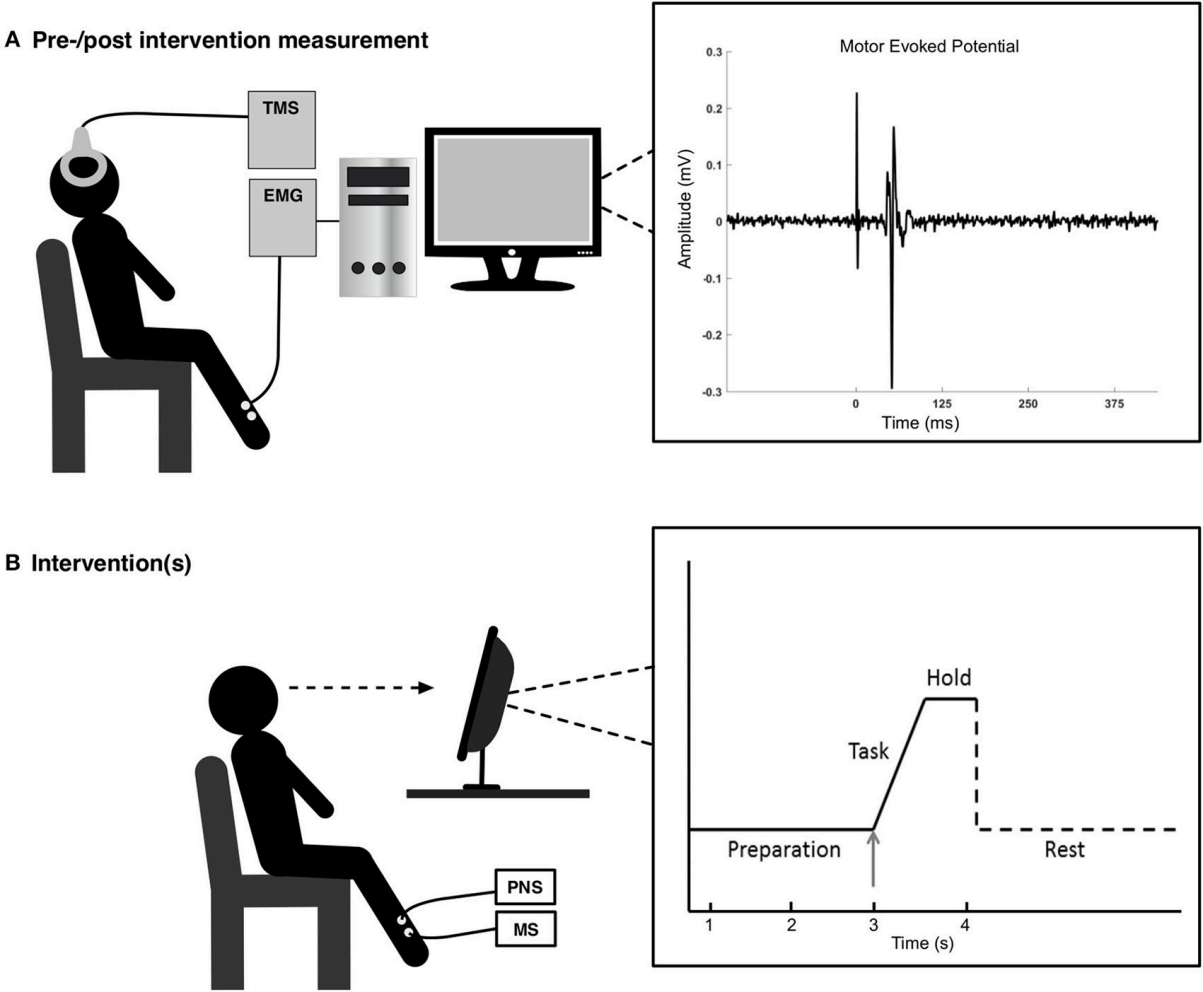
**In the top figure (A) to the left, the experimental setup is shown for the pre-/post-intervention measurements, and to the right is a motor evoked potential shown for a representative subject**. In the bottom figure **(B)** the experimental setup is shown for the interventions. The visual feedback to the subject (right part) consisted of 3 s of preparation before executing the task, a ballistic voluntary dorsiflexion of the ankle joint. They maintained the contraction for 1 s. Each movement was separated by ~10 s.

The subjects performed ballistic dorsiflexions of the right ankle joint; the movements were visually cued by a custom-made MATLAB program. The task can be seen in Figure 1B. The subjects had 3 s to prepare for the task, before they had to begin the movement (rise time of 100 ms) and hold the contraction for 1 s. A rest phase of 7 s followed the execution of the movement. The trace (solid lines in the right part of Figure 1B) was shown to the subjects with the arrow indicating the timing of the different phases. The subjects spend ~2 min familiarizing themselves with the task.

#### Electrical nerve stimulation

ES was applied to the deep branch of the right common peroneal nerve. A pair of surface electrodes (32 mm, PALS, Platinum, Patented Conductive Neurostimulation Electrodes, Axelgaard Manufacturing Co., Ltd., USA) was placed on the skin over the nerve with the cathode proximal and anode distal. The optimal recording site was defined as activity only in TA during stimulation without any activity in synergistic and antagonistic muscles. This site was confirmed by palpating the surrounding muscles. When the optimal site was found, the motor threshold (MTh) was determined as the lowest stimulation intensity that elicited a response in the TA tendon, as determined by palpating the tendon (Jochumsen et al., 2015). The pulse width was 1 ms and the stimulation intensity was fixed at MTh as described in a similar study (Mrachacz-Kersting et al., 2012). A Digitimer Stimulator DS7AH was used to deliver the stimulus.

#### Electrical muscle stimulation

ES was applied over the TA muscle. The surface electrodes were placed on the skin over the belly of the muscle with the cathode proximal and anode distal. The optimal recording site was defined as described above. The pulse width was 0.1 ms and the stimulation intensity was fixed at MTh. The stimulation frequency was 80 Hz. A NeuroTrac®Rehab ECS305A Dual Channel TENS and NMES Stimulator was used to deliver the stimulus. The lower pulse width was chosen to make the stimulation more comfortable than what was associated with a pulse width of 1 ms.

#### Motor-evoked potentials

Single pulse TMS was used to elicit motor-evoked potentials (MEPs) in the right TA with a Magstim 200 (Magstim Company, Dyfed, UK) using a figure-of-eight double-cone coil with a posterior-anterior current direction. The MEPs were recorded using electromyography (EMG) from the right TA. Surface electrodes (20 mm Blue Sensor Ag-AgCl, AMBU A/S, Denmark) were placed over the belly of the muscle. EMG was sampled at a frequency of 4000 Hz, and amplified and band-pass filtered from 20 to 1000 Hz, using a customized EMG amplifier.

### Experimental setup

The experiment comprised four conditions for all subjects: (1) nerve-located ES paired with voluntary dorsiflexion, (2) muscle-located ES paired with voluntary dorsiflexion, (3) voluntary dorsiflexion alone, and (4) muscle-located ES alone. The order of the conditions was randomized for each subject. Exposure to different conditions for the same subject was separated by at least 24 h.

The following procedure was similar for all conditions. During the session participants were seated in a comfortable chair (see Figure 1). After the ES sites were determined (except for condition 3), the optimal site for evoking MEPs in the TA was determined. This site was determined by measuring peak-to-peak values of the MEPs and the optimal spot was found where the peak-to-peak values were larger than the adjacent areas (Hallett, 2007). This hotspot was marked to ensure that the coil would be held in that position throughout the experiment. Once the optimal stimulation site was found, the resting threshold (RTh) was determined. This was defined as the lowest stimulator output where 5 of 10 stimuli elicited an MEP greater than 50 μV peak-to-peak amplitude. Before, immediately after and 30 min after each of the conditions, 12 stimuli were applied over the cortex at five different intensities: 90, 100, 110, 120, and 130% of the RTh, so 60 stimuli were applied in total. Twelve stimuli at one intensity is a block, i.e., five blocks were performed. The order of the blocks was randomized, but fixed throughout the experiment, so the same order of the blocks was performed before, after and 30 min after the intervention. Each stimulus was separated by 5–7 s. These five different intensities were chosen to obtain an input-output relationship between the stimulation intensity and size of the MEP by fitting a Boltzmann sigmoidal function.

### Conditions

Each of the four conditions consisted of 50 trials with a short break after 25 trials; individual trials were separated by ~10 s. In condition 1 the subjects were asked to perform voluntary dorsiflexion of the ankle (as described above) while nerve-located ES (over the CPN) was delivered 50 ms before the cued movement onset. This was to ensure the peripheral electrical stimulus reached the somatosensory cortex close to the onset of the movement. The 50 ms period was based on the average conduction time in the nervous system and cortical processing delays (Mrachacz-Kersting et al., 2007, 2012). In condition 2 participants were asked to perform voluntary dorsiflexion of the ankle while muscle-located ES was delivered 1150 ms before the task onset. It took 1100 ms from the activation of the muscle stimulator until a force response was obtained; this delay was determined in a pilot experiment involving five subjects. In condition 3 voluntary dorsiflexion alone was performed. In condition 4 muscle-located ES was delivered alone, with no voluntary movement. The stimulation in condition 4 was delivered with the same timing as in condition 2.

### Data analysis

Initially, the peak-to-peak value amplitude of each MEP was measured for all stimuli. The pre- and post-intervention stimuli were averaged for each TMS intensity and fitted with the Boltzmann sigmoidal function using the Levenberg-Marquard nonlinear least-mean-squares algorithm (Devanne et al., 1997). From this function a relationship was determined between stimulation intensity and peak-to-peak amplitude of the MEPs. The following parameters were extracted from the input-output relation of the sigmoidal fit: (i) the maximum peak-to-peak amplitude (MEP_max_) (ii) the intensity needed to obtain 50 % of the maximum peak-to-peak amplitude (S_50_), and (iii) the slope (K). The MEP_max_ is the maximal motor response that is obtained while the S_50_ and slope represent the threshold and gain, respectively, of the corticospinal neurons and motoneuron pool.

### Statistical analysis

The pre-intervention averaged peak-peak MEP_max_ values, S_50_ and slope were compared using three 1-way repeated measures analysis of variance (ANOVA) with “condition” as a factor (four levels: voluntary movement paired with nerve-located ES, voluntary movement paired with muscle-located ES, voluntary movement alone, and muscle-located ES alone). A 2-way repeated measures ANOVA was performed on the three parameters of the sigmoidal fit with the factors “time” (three levels: pre-intervention, post-intervention and 30 min post-intervention) and “condition.” Statistical significance for all tests was set at *P* < 0.05. Significant test statistics were followed up with a *post hoc* test with Tukey's correction to avoid errors associated with multiple comparisons. If the assumption of sphericity was violated the Greenhouse-Geisser correction was used.

## Results

Initially, a 1-way ANOVA was performed to test for differences between the parameters extracted from the sigmoidal fit in the pre-intervention measurements. The effect of “condition” was not significant for MEP_max_ [*F*_(3, 33)_ = 2.45; *P* = 0.081], S_50_ [*F*_(3, 33)_ = 0.78; *P* = 0.52], and the slope [*F*_(3, 33)_ = 0.35; *P* = 0.79] meaning that at baseline the parameters were not different across the conditions. The values of the sigmoidal fit are presented in Table 1.

**Table 1:** **Averaged values for the input-output parameters are presented for the four conditions**.

**Condition**	**Measurement**	**MEP_max_ Mean ± SD**	**S_50_ (% RTh) Mean ± SD**	**Slope Mean ± SD**
VM + N/ES	Pre	0.25 ± 0.21	99 ± 12	0.10 ± 0.040
	Post	0.27 ± 0.23	100 ± 12	0.17 ± 0.32
	Post 30	0.26 ± 0.17	87 ± 59	0.080 ± 0.040
VM + M/ES	Pre	0.19 ± 0.070	96 ± 16	0.090 ± 0.050
	Post	0.29 ± 0.13	99 ± 10	0.11 ± 0.080
	Post 30	0.29 ± 0.18	98 ± 11	0.18 ± 0.34
VM	Pre	0.30 ± 0.15	101 ± 9	0.10 ± 0.060
	Post	0.30 ± 0.23	98 ± 6	0.20 ± 0.27
	Post 30	0.29 ± 0.21	97 ± 11	0.19 ± 0.31
M/ES	Pre	0.32 ± 0.22	102 ± 16	0.080 ± 0.040
	Post	0.32 ± 0.30	99 ± 25	0.070 ± 0.040
	Post 30	0.38 ± 0.34	99 ± 12	0.11 ± 0.10

*VM, voluntary movement; N/ES, nerve-located ES; M/ES, muscle-located ES; post30, 30 min post intervention. The r^2^-value of the sigmoidal fit was on average 0.75 ± 0.23*.

Next, the effect of time and condition was quantified for the input-output parameters from the pre-, post-, and 30 min post-intervention measurements. For MEP_max_, the interaction between the two factors was significant [*F*_(6, 66)_ = 2.65; *P* = 0.023], but the effect of “time” [*F*_(2, 22)_ = 1.78; *P* = 0.19] and “condition” [*F*_(3, 33)_ = 0.90; *P* = 0.45] was not significant. The MEP_max_ increased significantly from pre- to post- (*P* = 0.0061) and pre- to 30 min post-intervention (*P* = 0.017) for paired muscle-located ES and voluntary movement. For S_50_, the interaction between the two factors was not significant [*F*_(6, 66)_ = 0.56; *P* = 0.76], nor was the effect of “time” [*F*_(2, 22)_ = 0.91; *P* = 0.42] and “condition” [*F*_(3, 33)_ = 0.68; *P* = 0.57]. For the slope, the interaction between the two factors was not significant [*F*_(6, 66)_ = 0.64; *P* = 0.70], nor was the effect of “time” [*F*_(2, 22)_ = 0.84; *P* = 0.44] and “condition” [*F*_(3, 33)_ = 1.05; *P* = 0.39].

The MEP_max_ increased from the pre- to post-intervention measurement with 50 ± 57 and 43 ± 55% for the pre- to 30 min post-intervention measurement when voluntary movement was paired with muscle-located ES. This was a higher percentage change from the pre-intervention measurement than the other conditions. The changes for the different interventions can be seen in Table 2.

**Table 2:** **The changes (%) from pre- to post-intervention and pre- to 30 min post-intervention measurement are presented for the input-output parameters for each of the four conditions**.

**Condition**	**Measurement**	**MEP_max_ (%) Mean ± SD**	**S_50_ (%) Mean ± SD**	**Slope (%) Mean ± SD**
VM + N/ES	Pre-Post	28 ± 54	2 ± 4	49 ± 212
	Pre-Post30	32 ± 69	15 ± 69	−15 ± 38
VM + M/ES	Pre-Post	50 ± 57	8 ± 36	−2 ± 170
	Pre-Post30	43 ± 55	7 ± 35	14 ± 235
VM	Pre-Post	−5 ± 28	−2 ± 8	66 ± 102
	Pre-Post30	−11 ± 25	−4 ± 8	177 ± 612
M/ES	Pre-Post	2 ± 42	−5 ± 19	−6 ± 51
	Pre-Post30	17 ± 48	−1 ± 20	33 ± 89

*VM, voluntary movement; N/ES, nerve-located ES; M/ES, muscle-located ES; post30, 30 min post intervention. A negative change means that excitability decreased from pre- to post- (or 30 min post) intervention*.

## Discussion

In this study, we investigated the effect of pairing voluntary movement with muscle or nerve-located ES. An increase in the excitability of the cortical projections of TA was only found for voluntary movement paired with muscle-located ES (condition 2).

### The effect of pairing electrical stimulation with voluntary movement

It was confirmed that pairing of voluntary movement with ES increases the excitability of the cortical projections of TA. The MEP_max_ increased 50 ± 57 and 28 ± 54% from pre- to post-intervention measurements in muscle-located and nerve-located ES, respectively. The increase in MEP_max_ outlasted the stimulation and was retained for 30 min for voluntary movement paired with muscle-located ES.

The increase in excitability was in the range of that reported previously, where an increase of approximately 30% from baseline was obtained using a similar protocol with single pulse nerve-located ES (Mrachacz-Kersting et al., 2012). However, the increase in MEP_max_ when voluntary movement was paired with muscle-located ES was not significantly different compared to nerve-located ES. The increase was in the range of that reported when pairing voluntary movement with repetitive ES. Previously, an increase of 66% was obtained when repetitive nerve-located ES was delivered during voluntary muscle contraction (Khaslavskaia and Sinkjaer, 2005). However, the duration of the stimulation needed to induce an increase in the study by Khaslavskaia and Sinkjaer (Khaslavskaia and Sinkjaer, 2005) was longer than that applied in the current study. In that study an increase was observed for muscle activity and ES as well. In the current study, however, there was no effect of voluntary movement alone, or ES alone, on the cortical excitability, potentially due to the low number of repetitions. In general, the standard deviations obtained for the changes from pre- to post-intervention measurements are relatively high, but in the range of what has previously been reported for similar protocols (Mrachacz-Kersting et al., 2012; Niazi et al., 2012). Different factors have been identified to account for some of the intra- and inter-subject variability such as: attention, age, time of day, brain anatomy (the TA representation lies deep in the interhemispheric fissure), gender, and genetics (Ridding and Ziemann, 2010).

The difference in excitability that was obtained between the two interventions where voluntary movement was paired with ES may be due to the afferent feedback that reaches the somatosensory cortex following muscle-located ES vs. nerve-located ES. The modulatory effect on the excitability from afferent feedback may be changed by varying the parameters of the ES such as frequency (Mang et al., 2010) and stimulation intensity (Chipchase et al., 2011). In both interventions the stimulation was delivered at MTh, but due to the frequency of muscle-located ES the afferent volley is different potentially due to temporal summation. An increase in excitability was obtained even though the stimulation intensity was rather low. A larger effect may be obtained by using higher stimulation intensities (Chipchase et al., 2011), but the best stimulation parameters to use have not been investigated thoroughly. The significant increase in excitability outlasted the stimulation and 30 min after, but it is not known for how long the increase was maintained. In previous studies, the increase in excitability was maintained between 30 and 120 min for different stimulation protocols using ES, ES paired with a cortical drive and PAS (Hamdy et al., 1998; Khaslavskaia et al., 2002; McKay et al., 2002; Charlton et al., 2003; Knash et al., 2003; Khaslavskaia and Sinkjaer, 2005; Mrachacz-Kersting et al., 2007).

### Possible mechanisms

The underlying physiological mechanisms may be mediated through LTP-like mechanisms, with alterations in synaptic efficacy. The most likely candidates responsible for the rapid changes in the excitability of the sensorimotor cortex are: modulation of activity-dependent synaptic plasticity, activation of silent synapses (possibly through up regulation of AMPA receptors on the postsynaptic membrane), unmasking of latent horizontal connections, increased release of excitatory neurotransmitters, changes in synaptic weight and reduction of GABAergic inhibition (see Thickbroom, 2007 for a review).

Some criteria have been linked with LTP-like mechanisms such as: rapid onset, persistence on cessation of stimulation, associativity, specificity and NMDA-receptor dependence. All these criteria have been confirmed in PAS studies (Stefan et al., 2000, 2002; Wolters et al., 2003), supporting the view that in PAS interventions LTP-like mechanisms are responsible for the cortical excitability changes. In this study, three of the five criteria were fulfilled; rapid onset, persistence on cessation of stimulation, and associativity. Specificity and NMDA-receptor dependence were not investigated. In a similar study, specificity was explored and it was found that imagined dorsiflexion paired with ES at the CPN only led to an increase in TA MEP, with no increase in cortical excitability of the antagonist muscle, soleus (Mrachacz-Kersting et al., 2012).

The origin of changes in cortical excitability was not tested in this study. However, this has been investigated in several previous studies. In particular, it has been investigated whether the changes in excitability are observed only at a cortical level, or if changes are observed throughout the nervous system. Techniques such as F-waves, H-reflexes, and stretch reflexes have been applied to determine excitability of spinal motoneurones. Electrical brain stem stimulation has been used to investigate the descending excitability of the corticomuscular system from the cortex. Following interventions using PAS (Stefan et al., 2000; Wolters et al., 2003; Kumpulainen et al., 2012), ES (Ridding et al., 2000; Khaslavskaia et al., 2002; Knash et al., 2003; Thompson et al., 2006) and pairing of motor imagery with ES (Mrachacz-Kersting et al., 2012), it has been found that the origin is likely to be supraspinal or cortical, however, spinal contribution to the excitability changes should not be ruled out due to the limitation of the different techniques.

### Limitations

A limitation of the study was that the MRCP was not recorded, so it is not known if the ES was delivered with the correct timing according to peak negativity (Mrachacz-Kersting et al., 2012). There is a possibility that some of the 50 trials were paired with an incorrect timing, but we believe that healthy subjects can perform movements to a visual cue consistently. From a clinical point of view, however, stroke patients may have difficulties in performing movements consistently according to a cue. Therefore, another approach may be needed where peak negativity of the MRCP is detected in real time from continuous EEG recordings (Niazi et al., 2011; Xu et al., 2013; Jochumsen et al., 2015a,b), and ES is triggered based on this (Niazi et al., 2012). In this scenario, the risk for the patient timing the movements incorrectly will be reduced, and it will be possible for the patient to control the pace. Some patients may have difficulties in performing fast movements, so it should be considered to perform motor execution according to the ability of the patient; however, it has been shown that stroke patients can perform relatively fast movements, and that these can be detected from the EEG (Jochumsen et al., 2015a). For the electrical stimulation, the optimal stimulation site could be determined by EMG recordings; this could have given a more precise estimate of the muscle activation; however, it has been shown previously that manual palpation is reliable (Bertelli, 2015). Lastly, it should be noted that the number of subjects in this study was limited, and that only healthy subjects participated. Thus it would be interesting to investigate if similar changes are observed in people with motor impairment after, e.g., stroke, and if they can lead to functional improvements.

### Conclusions and implications for stroke rehabilitation

The excitability of the cortical projection of TA increased when muscle-located ES was paired with voluntary movement. These findings suggest that muscle-located ES should be paired with voluntary effort for neuromodulation protocols to have maximal potential to increase cortical excitability and improve patient outcomes. Also, by using muscle-located ES, it will be possible to replicate different movements and in this way introduce task variability in training protocols. By introducing task variability (using different movements), the retention of motor learning may be maximized and the generalization of learning new movements may be optimized (Krakauer, 2006). This could be useful in neurological rehabilitation to enhance recovery of motor function. Further studies are needed to optimize the stimulation parameters and investigate the efficacy of the protocol in people with stroke.

## Author contributions

MJ, IN, NS, KH, HH, and DT designed the work. MJ, IN, and RN collected and analyzed the data. All authors helped interpreting the results. MJ drafted the manuscript while all the other authors revised the manuscript critically. All authors approved the final version of the manuscript.

### Conflict of interest statement

The authors declare that the research was conducted in the absence of any commercial or financial relationships that could be construed as a potential conflict of interest.
